# Child-Friendly Legal Aid and Individual Assessment of Children in Conflict with the Law: Building the Basis for Effective Participation

**DOI:** 10.3390/ijerph19010017

**Published:** 2021-12-21

**Authors:** Agnė Limantė, Rūta Vaičiūnienė, Jolanta Apolevič

**Affiliations:** Law Institute, Lithuanian Centre for Social Sciences, Ankštoji g. 1A, LT-01109 Vilnius, Lithuania; ruta.vaiciuniene@teise.org (R.V.); jolanta.apolevic@teise.org (J.A.)

**Keywords:** children’s rights, child’s needs, child in conflict with the law, participation in the proceedings, hearing of the child, individual assessment, child-friendly legal aid, youth justice

## Abstract

This article focuses on the importance of the right to effective participation of children in conflict with the law in criminal (youth justice) proceedings. In particular, it explores two procedural aspects which are closely related to the right to child-friendly legal aid and the role of individual assessment. The authors claim that qualitatively identifying the needs of the child (through the instrument of individual assessment) and establishing a relationship of trust with a specifically trained legal aid lawyer is critical in raising the child’s status to that of an active participant in the proceedings and ensuring that the best interests of the child are observed. The authors also suggest that such an initial encounter within the justice system forms an attitude (of either confidence or rejection) of the child towards public institutions and, accordingly, directly affects the effectiveness of further interventions. To support their position, the authors of the paper bring several examples from the comparative research carried out in two European projects, noting, regretfully, that the initial interventions often suffer from formal implementation and lack a systemic approach.

## 1. Introduction

The legal expressions of ‘juvenile defendant’s participation in the proceedings’ and ‘hearing of the child’ in criminal (youth justice) proceedings carry a considerably different content than their basic linguistic meaning. It is far from sufficient just to admit the young defendant to the courtroom, to provide some time to express his (her) position and explain himself, or to allow him to get acquainted with the documents or permit challenging them. The concept of a child per se means that we are dealing with a person who is immature physically and psychologically, and who is thus much more vulnerable (more traumatized and stressed about his (her) position confronting public institutions) than an adult in a similar situation. Indeed, children who end up in conflict with the law are among the most vulnerable in society [1]. Moreover, in most cases, children have limited knowledge of the complexities of the legal proceedings and legal implications imposed on their future, and they are less able to understand the peculiarities of specific actions taken against them and the importance of stepping out to participate in the proceedings effectively.

Based on these specific circumstances, we may instinctively come to a two-fold conclusion. On the one hand, children in conflict with the law need to be assisted in understanding criminal (youth justice) proceedings, expressing their views, and, in general, effectively participating in it. On the other hand, their specific situation should also be assessed and taken into account to enable the professionals involved to have more information about the child and his or her background. Individual assessment of the child and his or her situation allows the involved professionals to understand the child’s circumstances better and accordingly adjust the communication and decision making regarding the child. Otherwise, we risk that a child will not be seen as an individual with his (or her) specific experiences and need, but rather as a generalized and standardized object or “another problematic juvenile” defined in general terms. Avoiding this flawed practice, i.e., treating the child as a participant rather than a passive object, contributes to an effective response to his or her behavior, and improves the chances for successful implementation of the measures, including those related to the reintegration [2]. Furthermore, as noted in legal and criminological discourse, the child’s actual participation enables him to socialize within the legal system, build confidence in decisions, accept legitimacy, and trust the institutions that implement them [3]. It encourages the child offenders to develop a sense of responsibility, and can improve their rehabilitation and reintegration back into their community [4]. Finally, it can positively impact the development of autonomy, increase self-confidence and self-esteem, and contribute to developing specific skills (such as opinion formation or reasoning) [5,6].

Although the child’s right of participation is enshrined in international instruments and national laws of European countries, the question of whether, and to what extent, these guarantees are actually implemented remains open. Participation is a complex concept that can include various issues at different stages of the process; however, measuring and attempting to weigh participation is even more difficult [7]. As a result, while it is widely acknowledged that effective and meaningful participation is critical for realizing children’s rights and ensuring their best interests, protecting and empowering children, and improving decision-making and legal proceedings, there is still a gap or tension between theory and theory-in-use, and children are still rarely given a voice in reality [8].

Guided by such understanding, we seek to conceptualize the effective participation of a juvenile defendant in the proceedings against him or her, bringing specific elements of this ‘umbrella right’ into readers’ attention. Having analyzed the international instruments that safeguard the procedural rights of children in conflict with the law and the existing academic research, the paper then explores two procedural rights, which are vital to ensure the child’s best interests and his or her effective participation rights. In particular, the article focuses on the necessity of child-friendly legal aid and the role of individual assessment as tools to facilitate child’s participation and, in a broader sense, child-friendly justice.

In our analysis, we seek to reveal how, and to what extent, the legal aid and the instrument of individual assessment contribute to ensuring child’s participatory rights. We also aim to show practitioners’ perspectives on this issue. Moreover, using empirical data based on interviews with practitioners working in the child justice field, we attempt to identify and highlight the difficulties of putting these instruments into practice. Our main goal is, thus, to demonstrate that additional efforts should be put into addressing these issues. Otherwise, the child’s right to effective participation in the proceedings would be jeopardized.

A brief note regarding terminology should be made here. For the purpose of this article, the definitions ‘child in conflict with a law’/‘juvenile’/’minor’/’young defendant’ means a person who has reached the age of criminal responsibility but not the age of majority (under 18 years old), who is suspected or accused of having committed an offence under his or her national criminal law. (CRC/C/ GC/10, Introduction, §1) The age that needs to be taken into consideration to determine whether a child is in conflict with the law is no later than the age at the time of committing the offence.

## 2. International Legal Instruments Guaranteeing Juvenile Defendants’ Participation Rights

Following the adoption of several United Nations documents, a solid legal basis for the protection of minors in criminal proceedings was established. For the first time, the concept of participation in youth justice proceedings was introduced in 1985 in the Beijing Rules [9]. It was a soft law instrument sketching the model for national youth justice systems. In its rule 14.2, the Beijing Rules declared that the proceedings shall be conducive to the best interests of the juvenile and shall be conducted in an atmosphere of understanding, which shall allow the juvenile to participate therein and to express herself or himself freely.

However, it was the year 1989 that elevated the issue of children’s rights to a new level and brought it into the focus of the international community. As notes Liefaard, the UN Convention on the Rights of the Child can be regarded as a game changer, at least from an international human rights perspective [10]. With the adoption of the United Nations Convention on the Rights of the Child [11], a legally binding international instrument, the international community moved away from the child being perceived merely as a vulnerable and dependent human being, and began to recognize him or her as a full-fledged rights bearer. Equal to others, yet with a greater need for protection.

Article 12 of the UN Convention on the Rights of the Child formulated the child’s right to be heard which forms the essence of effective participation. As Rap notes, the right to be heard, as enshrined in Article 12, has significant implications for how juvenile defendants are treated in juvenile justice proceedings [12] (p. 94). Article 12(2), which provides that children should be given ‘the opportunity to be heard in any judicial and administrative proceedings affecting the child’, can be interpreted as a requirement to ensure the participation of juvenile defendants in the proceedings against them. Child participation rights are also established in Article 40(2)(b)(iv), which provides that a child alleged as or accused of having infringed the penal law have a right to examine adverse witnesses and to obtain the participation and examination of witnesses on his or her behalf under conditions of equality.

As explained in the General Comment No. 12 (2009) on the right of the child to be heard [13], the context in which a child exercises her or his right to be heard has to be enabling and encouraging, so that the child can be sure that the adult who is responsible for the hearing is willing to listen and seriously consider what the child has decided to communicate (para 42). Moreover, the right to be heard has to be fully observed during all stages of the judicial process, from the pre-trial stage when the child has the right to remain silent, to the right to be heard by the police, the prosecutor, and the investigating judge. It also applies through the stages of adjudication and disposition, as well as the implementation of the imposed measures (para 58). It is also noted that, to effectively participate in the proceedings, every child must be informed promptly and directly about the charges against her or him in a language she or he understands, and also about the juvenile justice process and possible measures taken by the court. The proceedings should be conducted in an atmosphere enabling the child to participate and to express her/himself freely (para 60). Overall, the hearing of the child carries material importance; to properly ensure the best interests of the child, the child should be heard in the case.

From the more recent documents, it is necessary to mention the UN Committee on the Rights of the Child General Comment No 24 “Children’s rights in juvenile justice” [2], which focuses on children alleged as, accused of, or recognized as having infringed criminal law. This document reminds us again that the child’s right to be heard is fundamental for a fair trial. He or she has the right to be heard directly, not only through a representative or an appropriate body at all stages of the process, starting with the pre-trial stage when the child has the right to remain silent. Furthermore, no adverse inference should be drawn if he or she elects not to testify throughout the stages of adjudication and implementation of the imposed measures. The comment also makes a logical conclusion that a child who is considered to be criminally responsible should be regarded as competent to effectively participate in all aspects of the trial (para 55).

These international principles and standards are reiterated and further strengthened by regional instruments. In Europe, the European Convention on Human Rights (ECHR) [14] and European Court of Human Rights (ECtHR) case law has been used to build the framework for ensuring minors’ rights in Europe. Of particular importance is the case law of the ECtHR interpreting Article 6, which provides a number of fair trial guarantees.

As explained by the ECtHR, in the case of a juvenile defendant, the right to effectively participate in his (her) criminal trial requires that the authorities deal with him with due regard to his vulnerability and capacities from the first stage of his involvement in a criminal investigation. The authorities must take steps to reduce the child’s feelings of intimidation and inhibition and to ensure that he has a broad understanding of the nature of the investigation and the stakes, including the significance of any potential penalty as well as his rights of defense, and, in particular, his right to remain silent [15] (para 195). Moreover, the right of an accused under Article 6 of the Convention to participate effectively in his or her criminal trial suggests that the court not only includes the right to be present but also to hear and follow the proceedings [16] (para 123). The “effective participation” also presupposes that a defendant, if necessary with the assistance of, for example, an interpreter, lawyer, social worker, or friend, should be able to understand the general thrust of what is said in court. The defendant should be able to follow what is said by the prosecution witnesses and, if represented, to explain his or her version of events to the defense counsel, point out any statements with which he or she disagrees, and make the trial court aware of any facts which should be put forward for the defense [16] (para 124).

Furthermore, as regards the best interests of the child, the court has held that, in the case of juvenile defendants, the criminal proceedings must be organized as to respect the principle of the best interests of the child during trial proceedings. It is essential that a child charged with an offence is dealt with in a manner that fully takes into account his or her age as well as their level of maturity and intellectual and emotional capacities, and that steps are taken to promote his ability to understand and participate in the proceedings [17] (para 85–86). This guiding principle is often reiterated in the case law.

In the European context, we should also mention the Council of Europe 2010 Guidelines on Child-Friendly Justice [18]. These guidelines cover and reflect all of the principles established in the UN CRC. Though they are not legally binding, they have served as a comprehensive and specialized set of practical tools for the Council of Europe’s forty-seven member states, encouraging them to take significant steps toward ensuring that justice processes, including those in the criminal justice system, consider the unique needs of children. The guidelines arguably provide probably one of the most extensive accounts of how child-friendly justice should be defined and substantiated in national juvenile justice systems [12] (p. 98).

The guidelines define that “child-friendly justice” refers to justice systems which “is accessible, age-appropriate, speedy, diligent, adapted to, and focused on the needs and rights of the child, respecting the rights of the child including the rights to due process, to participate in and to understand the proceedings, to respect for private and family life, and to integrity and dignity”. Notably, the guidelines list participation as the first of the fundamental principles. They link participation to the right of children to be informed about their rights, be given appropriate ways to access justice, and be consulted and heard in proceedings involving or affecting them should be respected. This includes giving due weight to the children’s views, bearing in mind their maturity and any communication difficulties that they may have in order to make this participation meaningful.

Turning to the EU level, one should mention the Directive 2016/800 on procedural safeguards for children who are suspects or accused persons in criminal proceedings (i.e., the first binding document exclusively concerned with a single group of persons), or suspected or accused minors [19]. This directive lays down standard minimum rules pertaining to certain rights of children in conflict with the law. Its Article 16 (Right of children to appear in person at, and participate in, their trial) provides that the EU Member States shall ensure that children have the right to be present at their trial and shall take all necessary measures to enable them to participate effectively in the trial, including by giving them the opportunity to be heard and to express their views. The directive also guarantees the right to information (Article 4), a lawyer (Article 6), and legal aid (Article 18), and also requires an individual assessment of a child (Article 7) as well as other procedural rights.

Overall, judging from the documents discussed above, we may summarize by stating that the effective participation of the minor defendant in the criminal (youth justice) proceedings has been fully established on international and regional (European) levels in recent decades. There is no question that this right exists; the elements thereof are also identifiable. Nevertheless, the challenging question that remains open is how this right should be better secured in practice and how the states and professionals working within the national juvenile justice system can ensure that the child’s participation is effective and not only pro forma.

## 3. Challenges in Ensuring Children Participation in the Youth Justice Proceedings

As the documents discussed above reveal, the right to a child’s participation in justice proceedings is a highly complex notion. On the one hand, it encompasses informing children of the processes and their rights, explains the legal rules and practices, provides other assistance to children, and listens to the child (or the right to be heard). On the other hand, as Rap and Klep argue, effective participation does not only imply that it is sufficient to implement practical and technical conditions, such as informing, hearing, explaining, or assisting children, but that it is also necessary to ensure that all of these technical conditions are implemented in practice and that children feel heard [7]. Furthermore, the perception of the notion gets even more complicated when we take into consideration that, until the end of the 20th century, children were seen as “objects of law” rather than “subjects”. Their status was determined, and interventions were made by legal experts (judges, prosecutors, and probation officers) who were presumed to know what was best for the child. The belief that children are incapable of dealing with certain “adult rights” persists to this day [3]. This flawed belief, along with the other key factors, such as the relative novelty of the concept of child participation, and limited development of this right, have contributed to the status quo when many challenges in implementing child participatory rights properly persist.

The first challenge is rooted in the aforementioned complexity of the notion of children participation. There are several definitions of children’s participation, such as participating in an activity or process that is more related to the practical and technical conditions (such as how children are informed about the procedure and whether or not they receive legal assistance), and participating in decision-making, which implies measuring the outcomes of children participation. However, as said before, measuring whether the child’s opinions have actually influenced the decision is a complex and challenging issue to solve [7,20]. For instance, the question of what is the “proper” weight to be attributed to the child’s views so it is in line with their interests remains unsolved. Various research findings reveal that the children’s views and wishes are only considered if the authorities agree with those views. Furthermore, there is minimal clarity about how and whether the child’s views influenced the final decision in most legal proceedings since children were rarely given feedback on how their opinions were taken into account during the decision-making process. Greater transparency in decision-making could be achieved by granting children autonomy and allowing them to choose whether and how they want to be involved unless it is likely that their wishes will result in significant harm [6,8,21].

Children’s influence on decisions is strongly linked to the concept of children’s capacities, age, and maturity levels, as stipulated in Article 12 of the UN Convention on the Rights of the Child. Professionals working in the field believe that, as a child grows older, their opinion is given more weight in decision-making. Furthermore, they emphasize that parents may be the primary impediment to children’s participation at times because they may have various reasons to prevent children from participating [7]. As a result, younger children and those who go against the prevalent norms (such as refusing contact with a parent) are disproportionately excluded [21]. Participation can only be interpreted in this context as adhering to the rules of a game that adults have already defined [3].

The second challenge—surprisingly as it might seem—involves a very well-developed discourse on child protection, which is a critical prerequisite for developing any international or national children’s rights policy. There is no doubt that the primary goal of this policy is child protection; however, this protection may sometimes come at the expense of the child’s participation. The discourse of children as victims who require adult protection affects professionals’ approaches and encourages them to doubt children’s capacities and decide for them to protect them [5]. Furthermore, some studies claim that professionals believe that children lack the necessary skills and competencies to participate and should be protected from participation. Besides, they are convinced that the effort required to have the child participate does not outweigh the benefits of participation [6]. However, the critics of such exclusionary approaches argue that participation does not imply that children require more authority and influence, but rather that their opinions must be considered alongside those of others [5]. As a result, reconciling the state’s duty to protect children while preventing paternalistic protected participation remains challenging [3].

The third problematic issue worth highlighting is a lack of expertise and time regarding the professionals working in the field of social welfare and youth justice. According to the research, some professionals lack the communication skills needed to talk with a child, or are accustomed to making assumptions about children’s needs and what is best for them. In addition, the widely accepted subordinated position of children does not allow for children to be seen as partners in decision-making and planning for children’s well-being [5]. Furthermore, due to the professional workload, professionals are not given adequate time to establish a relationship and trust [7]. Finally, organizational cultures are preoccupied with quantifiable targets and tasks, such as completed investigations, over those less quantifiable, such as building relationships and talking with children [8].

Another complex issue is that, regretfully, the youth justice system is currently inextricably linked to quantification, standardization, and objectification of decisions and judgments, which is supposed to be accomplished through the use of modern assessment and screening tools and instruments. Justice systems are obsessed with the language of ‘risk,’ which promotes a deficit-focused, reductionist view of children and their behavior, requiring them to accept responsibility for their actions [8,22]. The use of these instruments de-professionalizes practitioners by removing their ability to make judgments based on an in-depth and unbiased evaluation of the problem and prioritizing more mechanistic risk assessment [8]. Risk factors are solely evaluated from a practitioner’s perspective, as opposed to a child’s self-assessment of risk and a child’s perspective on other important aspects of his life. As a result, youth disengagement is primarily caused by enforced, adult-centric, and practitioner-rated quantified factors. In such cases, children cannot recognize the benefits of assessment and intervention and do not become positively and actively involved in youth justice processes [22].

The abovementioned challenges demonstrate that there is still room for debates about promoting children’s participation. To begin, it is critical to shift the focus away from children’s elimination from the proceedings in order to protect them and to turn it towards children’s active participation, engagement, and weighing of their opinions. Second, enhancing professionals’ capacities and soft skills and focusing on in-depth evaluations of children’s situations remain essential. Furthermore, building trust-based interactions between children and professionals are vital. Finally, we can only hope that the situation will change, and that children will be heard and presented with feedback on how their opinion has influenced the final decisions.

## 4. Method

The two further sections of this article focus on legal aid and individual assessment of accused and suspected children as two procedure-related elements strengthening a child’s participatory rights. The analysis is based on the findings of two EU co-funded Justice Programme projects researching specific procedural aspects of criminal (youth justice) proceedings: “Legal aid for children in criminal proceedings: developing and sharing best practices” (LA Child; JUST- AG- 2019/JUST- JACC-AG-2019-802059) and “Procedural safeguards of accused or suspected children: improving the implementation of the right to individual assessment” (IA-CHILD; JUST-AG-2017/JUST-JACC-AG-2017-802059). The content of this paper, however, represents the views of the authors only and is their sole responsibility. The European Commission does not accept any responsibility for use that may be made of the information it contains.

The ongoing project “Legal aid for children in criminal proceedings: developing and sharing best practices (LA Child)” has been implemented in three European countries from 2020: Albania, Belgium, and Lithuania. This article embraces and uses the empirical data from qualitative research conducted in each partner country. In particular, it relies on the semi-structured interviews that were conducted in partner countries: in Albania, 12 interviews were conducted with attorneys, 2 with judges, and 1 with a prosecutor; in Belgium, 11 interviews were conducted with youth lawyers and 4 with professionals from a legal aid office; and in Lithuania, 8 interviews were conducted with advocates and 2 with prosecutors.

The empirical data for this article also originates from the second project, known as “Procedural safeguards of accused or suspected children: improving the implementation of the right to individual assessment (IA-CHILD).” The project brought together academics from four countries (Cyprus, Croatia, Greece, and Lithuania), and the empirical research was conducted using qualitative methodology between 2019 and 2021. Each country had between 6 and 29 legal and non-legal experts participate in semi-structured interviews and focus group discussions (Cyprus had 6 legal and non-legal professionals; Croatia had 29 experts, 17 non-legal professionals, and 12 legal experts; Greece had 12 experts, 6 juvenile probation officers, and 6 legal professionals; and Lithuania had 17 experts, 10 non-legal professionals, and 7 prosecutors).

Both projects used qualitative methodology, and respondents in each research were chosen on purpose. The projects’ researchers identified professionals working with children or participating in the policy making in the area. To reach the policymakers and experts in relevant public institutions, official written requests to the institutions were made, and, in some cases, the experts were contacted directly. To select relevant legal aid lawyers, psychologists, and other non-legal professionals, we overviewed the case law to identify this type of professionals appearing before the court, and also asked the legal aid boards or other institutions to recommend the professionals that appear in child cases more often. Snowball sampling (when respondents suggest some other respondents) was also used in both projects.

In terms of data collection, the researchers of both projects developed unified interview guidelines, and the same guidelines were used in all countries throughout each project. The guidelines were developed using existing literature, recent empirical studies, and the international framework for children’s rights. In the LA Child project, the Interview Guidelines included four blocks of questions: (1) “General questions”, mainly focusing on experience and professional background of the respondents; (2) “National legal aid system” which included questions on the regulation of legal aid (existing instruments, and existing procedures and standards) and the application of rules in practice; (3) “Dealing with the case” covering the questions on the appointment of the lawyer, lawyer’s contacts with the child, lawyer’s presence and his/her role, language used, and relationship with the child; and (4) “Prospects for improvement” where the respondents were asked to indicate the most relevant (in their opinion) obstacles and challenges when providing legal aid to children in conflict with the law and to suggest their ideas as to how to improve the provision of legal aid to children. Parts of the questions were formulated broadly to allow the professional to discuss particular topics and points that were not foreseen by the research team and that correspond to the reality of the field. In the IA Child project, two distinct versions of interview guidelines were developed: one for experts conducting individual assessments and the other for experts using assessments. The interview questions in both versions covered five major thematical blocks: (1) general information about the expert’s background and experience; (2) existing regulations and national practice on current assessment in criminal proceedings in a specific country; (3) perception of individual assessment (aims, assessed areas, specialist involved, results); (4) process of individual assessment (stage, procedures, sources of information, contact with the child); and (5) difficulties and challenges for an effective, child-friendly, engaging assessment.

In both projects, before the interviews, the research participants were acquainted with the purpose and procedure of the research, ensuring their anonymity. Researchers in each country followed the ethical rules applicable in their institutions. Thus, before the interviews in Lithuania, the researchers shared the interview guidelines and all information related to the informed consent with the participants. Then, both research projects’ participants confirmed their participation by signing informed consent or through electronic means.

In the LA Child project, interviews were conducted from August 2020 until October 2020. The average duration of the interviews with the participants was around one hour each. Most professionals were interviewed remotely using teleconferencing software to adapt to the safety requirements linked to the COVID-19 pandemic. Interviews for the IA Child project were conducted from August 2019 to November 2019 and lasted approximately for one hour on average. Interviews were made either directly meeting the persons or remotely using teleconferencing software.

In both projects, most of the interviews were audio-recorded and transcribed, and the findings were only used in a generalized and summarized manner. Each partner country developed its own data coding and analysis system. The researchers in each country had certain flexibility and followed the procedure standard for their country. For instance, in Lithuania, each interview was assigned a code. The first letter indicated the first letter of a person’s profession and the number indicated the number of the interview. All the interviews were recorded and transcribed. Meanwhile, data analysis was carried out using the MAXQDA software, following a qualitative content analysis approach.

In this paper, we use primary data from interviews conducted in Lithuania and secondary data from national reports based on interviews conducted in other countries. Because all interviews in both projects were conducted in national languages, the authors of this paper were only able to directly analyze the data from interviews conducted in Lithuania. To prepare this particular paper, we additionally conducted an analysis of interview responses related to the effective children’s participation in criminal (child justice) proceedings. Such tactics were determined by the fact that, in both projects, children’s participation was not per se the primary goal of the research; this question was covered amongst other questions on the broader list. A systemic analysis of the rest of the topics covered during the interviews as well as a detailed presentation of the LA Child and IA Child researches can be found in Limante et al.’s “Legal aid for children in criminal proceedings: report on current European national frameworks (LA CHILD project report)” [23] and Vaičiūnienė’s “Individual Assessment of Suspected or Accused Children: Insights into Good Practice in the Light of the Directive (EU) 2016/800” [24]. It should also be noted that, in order to ensure that ethical standards are met, only the responses of the interviews conducted in Lithuania are cited directly in this paper (the researchers of the Law Institute of the Lithuanian Centre for Social Sciences have conducted the interviews; in respect of that the authors possess all the supporting documents). The data of other countries’ interviews are summarized, relying on the national reports.

There are certain limitations of the research paper that should be noted. Firstly, not all the persons that were identified by the researchers’ team as the most relevant respondents agreed to participate in the research. The significant obstacles were encountered when finding the qualified persons for the interviews, which is explained by the limited availability of juvenile justice professionals who generally have a busy schedule and, in addition, overall reluctance to take part in interviews that are conducted for the purposes of research. As a result, the views of some key people in the field might not have been heard. Secondly, to get acquainted with the interviews conducted in Belgium, Albania, Croatia, Greece and Cyprus we had to rely on the interview reports drafted by the researchers from those countries. As a result, only summarised information was available to us (transcripts of the interviews were not translated to English). Thirdly, due to the limits of the article, we could not extensively discuss differences in legal systems and procedural specificities; however, such additional descriptive information can be found in the publications noted above.

It should also be noted that both studies did not intend to present generalized data, but rather to investigate participants’ perceptions, interpretations, and beliefs, while seeking a complete and comprehensive picture of the specific topic from their point of view. Therefore, participants’ anonymity was guaranteed, except when the experts requested otherwise.

## 5. The Importance of Legal Assistance for Effective Participation in the Proceedings

The right to a lawyer is one of the most apparent means (and a condition sine qua non) to assist the child in comprehending and effectively participating in the proceedings. Such a link is confirmed in both legal and psychological theories. Analyzing from the developmental psychological perspective and based on the earlier research findings, Rap underlined that adolescents are not fully able to participate in a (youth) court hearing without the assistance and support of adults, because they have a limited understanding of the meaning of the process and of the attitude that is expected from them in court. She, thus, concluded that juveniles need additional assistance [12]. From a legal point of view, the right to legal or other appropriate assistance, as argued by Liefaard referring to legal instruments and ECtHR case law, is one of the most crucial prerequisites for children accessing justice and an essential element of fair and child-friendly treatment [11] (p. 209).

In the EU, the particular role of the lawyer is underlined in the already mentioned Directive 2016/800 on procedural safeguards for children who are suspects or accused persons in criminal proceedings [19]. In its Article 6(2), the directive requires the Member States to ensure that children are assisted by a lawyer in order to allow them to exercise the rights of the defense effectively. It should also be noted that EU law provides for the right to legal aid in Article 47 of the Charter of Fundamental Rights of the European Union. EU directive 2016/1919 on legal aid regulates the right to legal aid [25] in more detail and sets its quality standards. Moreover, we should note that, in its case law, the ECtHR has directly linked the right to a lawyer and a right to effective participation in the proceedings. In particular, it has been recognized that the de facto lack of legal assistance for most of the proceedings may exacerbate the consequences of the applicant’s inability to participate effectively in his trial and infringe his right to due process [16] (para 132). Finally, the importance of the assistance of a lawyer seeking to ensure procedural rights and best interests of children was also pointed out in recent comparative researches into national laws and practices in Europe [23,26].

Since children usually receive no income, in most situations, they cannot afford to hire a lawyer to defend them (and often the parents or caretakers are not able or willing to assist); thus, their right to legal aid becomes crucial. In the European continent, legal aid for children in conflict with the law is typically regulated, giving special attention to it in national laws on criminal proceedings and related instruments. Often, states establish a mandatory defense for children and do not apply means tests. For instance, in Lithuania, according to the Code of Criminal Procedure, a lawyer’s participation is required in the examination of cases where the suspect or accused is a child (all of such cases require mandatory defense). Children in conflict with the law are entitled to secondary legal aid regardless of their assets and income. Similarly, legal assistance is entirely free of charge for children in Belgium, Spain, France, Hungary, and many other countries [23].

However, as with most child-orientated measures, legal aid provided to children has to be adjusted to their needs. To say in other words, it should be child-friendly. The importance of children being provided with child-friendly legal aid has recently gained attention in both international and regional legal instruments [27]. For instance, the 2012 United Nations Principles and Guidelines on Access to Legal Aid in Criminal Justice Systems [28] set global standards for legal aid, and affirmed that legal aid should be accessible, effective, sustainable, and credible. This document gave considerable attention to children’s right to legal aid. Importantly, it defined the concept of “child-friendly legal aid”, noting that such legal aid should be accessible, age-appropriate, multidisciplinary, effective, and responsive to the range of legal and social needs faced by children and youth.

The importance of child-friendly legal aid in guaranteeing effective participation rights was also emphasized by the professionals that were interviewed during the LA CHILD project in Albania, Belgium, and Lithuania. They also noted the hurdles that limit the possibilities to provide quality legal aid for children.

The interviews with lawyers in Lithuania revealed that the earlier experiences of children in conflict with the law often determine the fact that these children, for whom the appointed defense lawyers provide their services, feel insecure and misunderstood, and have a negative attitude and no belief in themselves or the system. As a result, representing children urges for delicacy, attention, comprehension, and patience. As interviewers noted, it is often challenging to establish first contact with a represented child who is cautious when communicating with adults and expects to be blamed and not trusted. Honesty, sincerity, attentive listening, as well as a calm and friendly tone are the most important strategies to get in contact with a child, as claimed by one of the interviewers: “It is always necessary to smile at the beginning of the conversation and not to start it with what he has done” (Advocate 2, Lithuania). Moreover, interviewed lawyers noted that, during the interaction, it is important to make the children feel safe: “To show your attention, speak calmly and honestly, try not to push your beliefs or opinions for them or press them” (Advocate 2, Lithuania). The interviewed lawyers also highlighted that establishing communication with the child and encouraging them to take part in the proceedings is also essential to ensure the child’s effective participation in the proceedings. Sometimes, children withdraw into themselves, refrain from communication, deny everything, and either avoid or are afraid to testify; in such cases, the advocates must be particularly proactive:

“So, I say: “How do we look before the court if you arrogantly say that you will not testify?” Then I add: “Let’s behave differently”. I explain it to him very calmly and he testifies”.

(Advocate 4, Lithuania)

The interviews conducted in Belgium [29] also underlined the importance of the legal aid lawyer in enabling the child to participate in the proceedings effectively. The lawyers listed several roles they saw as an important part of their services, including advising, informing (on the procedure, on the child’s rights), listening, helping, guaranteeing the respect of rights, pleading for the child before the judge, and emphasizing their role as spokespersons for the young person. Like Lithuanian lawyers, Belgian lawyers stressed the need to build trust when working with children, which was seen as an essential step in advising and representing the child properly. This is achieved by employing different strategies, such as using child-friendly language, wearing non-formal attire, showing affection, and bringing trained dogs to the interview to reduce pressure.

In Albania, the interviews revealed that the lawyers believe it is their responsibility to assist the child during the interviewing session by ensuring that the child fully understands his or her rights and that the child interviewing process is carried out with several legal requirements (free of psychological pressure and misleading or incorrectly asked questions), and by intervening whenever the best interest of the child are not respected. Even though the interviewed professionals acknowledged that logistical and other flaws complicate communication with the child in some cases when ensuring his or her effective representation, the approach of the interviewed lawyers was orientated towards willingness to assist the child in protecting their rights. The importance of a trust-based relationship was again underlined, noting that the lawyers try to develop their own ice-breaking strategies as they talk with children.

It can thus be seen that the legal aid lawyers’ duty to assist the child in expressing his or her opinion, understanding the procedural particularities, and, in general, effectively participating in the criminal (youth justice) proceedings is not only established in international instruments and national laws or acknowledged in academic studies, but is also grounded in the approaches of (at least part of) legal aid lawyers. The lawyers do seek to build trust-based relationships with their young clients, communicate with them in a child-friendly manner, and, in this way, become spokespeople for children and guarantors of their rights. If the lawyers adhere to a child-friendly approach, provide a child-friendly legal aid, understandably explain the procedure, encourage the child to speak, hear the views of the child, help the child to express themself before a prosecutor and a judge, and ensure that the bests interest of the child are observed, the child’s right to participate in the proceedings effectively will be protected.

Despite this, in practice, many flaws exist that negatively affect lawyers’ ability to provide child-friendly legal aid. They are related to many factors, such as the attitude of the professionals involved, their lack of specialization, as well as practical challenges of the work.

Firstly, some lawyers still consider that older children (16–18-year-olds) in conflict with the law are mature enough to be treated as adults. One research participant who viewed older children as mature enough even wondered if a psychologist is really necessary to communicate with a child:

“If a young person is seventeen years old, maybe there is no need to interrogate him in a separate room with a psychologist? Maybe the judge could interrogate such individuals directly and then decide on the need for a psychologist’s help in such a situation?”

(Advocate 3, Lithuania)

On the other hand, most of the lawyers emphasised that the psychologists play the most important role: “They interact in a pleasant setting that reminds the nursery. It is achieved by playing there in the form of a game. On the other hand, there most of the lawyers emphasise that the psychologists play the most important role: “They interact in a pleasant setting that reminds the nursery. It is achieved by playing there in the form of a game. The psychologist, who has the proper education, spends an exclusive time with the child and is the only one who interacts. Then, based on his knowledge and experience, he formulates a judge’s question for a child in a way that the child could easily understand, and receive all answers. Later, everything is documented and read” (Lawyer 4, Lithuania).

Secondly, in some cases, we still confront the stigma that juvenile delinquency is linked to socially disadvantaged families who lack social skills. In such a situation, prejudices are expressed against children, which affect a lawyer’s work.

In my practice, there are more cases of secondary legal aid because our children (i.e., the suspects) usually come from poor families or families with limited social skills. A rare child comes from a “normal” family. Perhaps I used a wrong expression, but children who come from families that have never dealt with the State Child Rights Protection Service, from the families who have no problems and are not considered risky, etc.—rarely commit crimes. More often, such children commit disciplinary offences, etc.

(Prosecutor 2, Lithuania)

“It is such a group of people who are probably not too interested in their children; therefore, they are not interested in their rights or obligations either. This is misfortune. That is why it happens later on. Because children miss attention and control”.

(Advocate 3, Lithuania)

However, if the child does come from a disadvantaged family with a lack of social skills, they will likely need more assistance than other legal aid clients. Representation of such children requires even more delicacy, comprehension, and patience. Communicating with the adults and trusting them could be especially hard for children from complicated families; therefore, prejudices further enhance a child’s vulnerability. Similar hurdles (plus challenges arising from cultural differences) can occur in case of children who are asylum seekers or illegal migrants.

Another serious obstacle to child-friendly legal aid is the lack of specialization of legal aid lawyers. In some European countries, such as in Belgium, Finland, Albania, Italy, and France, to provide legal aid to children, a lawyer has to be on the “youth lawyers list”, i.e., to be specialized in providing legal aid to children. However, there is no such special category of “youth lawyers” in some countries. The lawyers provide legal aid to children and adults, and no delimitation of specializations with regard to the age of the legal aid recipient is made. This is notably the case for Lithuania, Germany, Austria, Czech Republic, Ireland, and Poland. Specialization, however, is critical due to the specific training gained. A large number of professionals interviewed in Albania, Lithuania, and Belgium during the project believe that working with children in conflict with the law requires specific skills and special knowledge (see above). The professionals interviewed emphasized the need to have a good understanding of the procedures applicable to children but also to have knowledge on the philosophy of youth law, which is very different from criminal law for adults as it is protective and not repressive. They need to be well informed on topics regarding adolescence and delinquency, and, moreover, soft skills of communication with children as well as specialized knowledge of psychology are needed [23,26]

Furthermore, some practical issues can influence lawyers’ ability to provide child-friendly legal aid. In Belgium, the interviewed professionals highlighted that the children and their lawyers cannot always discuss the case in a place that ensures confidentiality, as problems with confidential premises arise in some police stations and courthouses. This was reiterated by Albanian professionals who stated that the interviewing facilities at the police station, prosecution office, or court are not child-friendly. Thus, there is no specific room for interviewing children. Moreover, during the interview, other police officers that are not related to the case can walk into the room. This conflict with the principle of confidentiality discourages the child from talking and disclosing sensitive details about the event. Additionally, in Albania, during the legal session at the court, the juvenile stays behind the barriers and is distant from the lawyer and the psychologist.

Finally, the heavy workload of legal aid lawyers and limited time that a lawyer can dedicate to a child might influence their ability to devote needed attention to the child and allow time to build contact, explain the legal issues clearly, or keep constant contact with the child.

However, I would just like to mention another significant element. Since I meet and drop a word with legal aid lawyers regularly, I do know that these people are insanely busy. I think that their workload is too intense for them to be able to dedicate themselves completely to each case and do their work the best they can. Since they rush from one hearing to another, their schedules are stuffed and cramped with these cases; they have to be at one end of the city for a moment and hurry to another city for another moment. They hurry from one court to another, as they don’t get a fair payment for secondary legal aid, as far as I am aware. (Prosecutor 1, Lithuania) The workload was also mentioned during the interviews in Belgium. Such workload is further increased by administrative burdens.

Limited time to communicate with the child was also underlined by Albanian professionals who stated that the time to talk with the juvenile before the police interview is not enough (in Albania, the lawyer usually can meet the child for about 10 min before the interview, sometimes even this time is not given). In Belgium, lawyers similarly mentioned difficulties in contacting their young clients and, in some cases, regretted having to meet them for the first time only in court. Naturally, this seriously hinder lawyers’ ability to provide child-friendly services.

## 6. Individual Assessment of Children as a Tool to Encourage and Enhance Their Participation

As noted earlier, the Directive 2016/800 on procedural safeguards for children who are suspects or accused persons in criminal proceedings [19] calls for juvenile justice to be tailored to the needs of children and encourages the child’s effective participation in the process. In addition to other novelties, the directive incorporates an instrument of individual assessment. In this way, for the first time, a binding EU document expressly takes into account various legal issues and objectives involving child’s special needs and identifying areas in which young people feel and are most vulnerable.

The individual assessment of suspected or accused children might be analyzed as an instrument with two purposes. First, it is an evidence-based decision-making tool, which enables the child’s needs to be satisfied at every stage of the criminal process. Second, it may aid in involving children, hearing the child, representing and interacting with him or her, evaluating their situation and viewpoint, and assisting in reaching child-friendly decisions.

By providing and evaluating detailed information about the child, including his living environment and situation, individual assessment could play an important role in strengthening the child’s right to participate in justice proceedings. In particular, the information collected during the individual assessment forms the basis for better understanding the child and why he or she appeared to be in conflict with the law. It provides information that—at least in an ideal situation—should be read and taken into account by all the professionals (lawyer, prosecutor, judge, psychologist, other relevant bodies). Individual assessment allows the professionals to figure out the most effective ways to approach the child and encourage his cooperation. From this perspective, the tool of individual assessment also helps to ensure child-friendly legal aid (discussed above) as the lawyer can learn more about the child, his environment, and personality.

However, the benefits of individual assessment become apparent only if it is adequately adopted in national law and its practical implementation takes into account the instrument’s purposes. Currently, however, it seems that, in some countries, a lot still has to be done to achieve this.

Directive 2016/800 sets basic principles, giving the Member States leeway in adopting and administering specific child assessment models in their national systems. Different paths of implementing individual assessment were revealed in the IA Child project research on implementing individual assessment in Cyprus, Croatia, Greece, and Lithuania. Individual assessments in Lithuania, for example, contain a summary of information about the juvenile’s personality; environment; and needs for protection, education, and social integration prepared by territorial units of the Child Rights Protection Service. Individual assessment reports are not interpreted or related to specific legal issues, and they cannot be regarded as expert conclusions. The different situation is observed in Croatia, where various assessments are conducted depending on the specific case, phase of criminal proceedings, purpose, objective, assessment issues, focus, assessment steps, and other elements. The final result of the various evaluations should be a written opinion and proposal. In addition, it should contain all relevant information and recommendations for making decisions or implementing interventions. The State Attorney’s Office, Youth Courts, and Centres for Social Welfare are tasked with conducting individual assessments during criminal proceedings. In Greece, juvenile probation officers are responsible for conducting a social inquiry, which is considered an individual assessment for children who have allegedly broken the law and submitting reports to judicial authorities. The juvenile probation officer assesses the child’s personality, family, and social environment; recommends the most appropriate measure or sanction/penalty; and assists judicial authorities in making appropriate decisions on the child’s case. Finally, in Cyprus, where there is a lack of comprehensive juvenile offender legislation, implementing individual assessments is extremely difficult and is done at the Director of the Police Force’s request. The report’s purpose is to be presented at the court hearing or before the hearing so that the judge(s) can decide on sentencing the juvenile offender or suspect. The assessment is based on information gathered by the Social Welfare Service officer at his or her discretion. There is no standardized procedure for evaluating juvenile offenders or suspects; instead, each professional relies on their skills and abilities as a social worker [24].

The different paths of implementing individual assessment, as presented above, impact the appearance of obstacles and challenges, not only in terms of completing particular assessment tasks but also in terms of involving children in the process. One of the issues identified in the research was the formalistic approach used by some countries to meet the directive’s requirements. Individual assessment, for example, is implemented in Lithuania in a rather formal manner, as explained by one of the research participants:

The state looked at this matter only in a formal way <…>. They merely duplicated the functions of the institutions; therefore, we will not get any actual benefit from it. As I told you before, we will collect the same information that we usually collect, i.e., the descriptive data from all other institutions.

(Prosecutor, Klaipėda District Prosecutor’s Office, Lithuania)

As noted, the institutions only provide actual, descriptive data about the child and information that they have about the child at the time. The Child Rights Protection and Adoption Service, to which individual assessment functions are delegated, explains how the set of information considered as individual assessment looks like:

Thus, they would describe what they see, mention the number of rooms, indicate whether a child has a separate space, bed or a writing-desk. Meanwhile, after visiting a family for the first time and have a single visit we will not be able to tell what kind of emotional bond is formed between a child and his/her parents.

(Specialist, The regional division of the State Child Rights Protection and Adoption Service, Lithuania)

The research participants pointed out that such an assessment would only reflect one of the directive’s objectives, which is to assess economic, social, and family circumstances by providing actual data on them. However, that would bring little additional value in the directive’s implementation in Lithuania because prosecutors previously obtained descriptive information on the child’s situation from different institutions. Furthermore, it appears that the law does not involve children when conducting individual assessments in such a formal manner.

Children’s disengagement in individual assessment appears to be widespread in the other countries studied. For example, research participants in Cyprus emphasized that in many cases, the Social Welfare Service officers consider a house visit unnecessary because they try to obtain previously gathered information from various sources [30]. In Greece, probation officers in charge of the assessment saw interviews with the juvenile and his or her family as the primary tool for developing a good profile of the child. However, they also noted that difficulties involving family and a child arise, as they are sometimes unwilling to cooperate with the Juvenile Probation Service [31].

According to the IA Child project research, difficulties in conducting assessments, involving children, and communicating with them occur in all countries, primarily due to a lack of qualified personnel or their excessive workload. As a result, there was a strong demand for training for law enforcement institutions and non-legal professionals in all four countries studied.

Another significant finding from the analysis is the importance of standardization in the assessment process to achieve more consistent and reliable assessments and improve the quality of subsequent decisions. Juvenile justice professionals emphasized the importance of adopting such instruments in countries where a lack of standardized, science-based tools was identified, such as Greece and Cyprus. Furthermore, there is no unified and structured methodology for conducting an assessment in these countries. Even in Croatia, where the holistic approach is observed, and a variety of standardized and non-standardized instruments are used (depending on assessment objectives, phase of criminal proceedings, focus, etc.), professionals advocate for a more harmonized and uniform system [32].

From the perspective of professionals working in the youth justice or (and) welfare fields in all four countries covered in the IA Child project (Lithuania, Cyprus, Greece, Croatia), there is a lack of instruments or unified guidelines for implementing individual assessments of accused and suspected children and how to involve or contact them in the process. As a result, experts believe that a single universal instrument or practice that provides concrete instructions and produces specific weight in court as evidence-based conclusions could provide a solution. However, first and foremost, competencies and understanding of how to ensure active, positive involvement and representation of young people and the application of the most personalized approach possible should be strengthened. Secondly, the immediate obstacles, such as real or perceived lack of resources, the sufficient number of qualified professionals, poor working conditions, and child-unfriendly working environments, must be addressed. Hence, one of the primary goals should be to raise awareness among policymakers, other stakeholders, and legal and non-legal professionals about the importance and potential of individual assessment as a tool for balancing evidence-based decision-making with children’s views and ensuring children’s participation in the process.

## 7. Conclusions

Analysis of international instruments guaranteeing children’s rights reveals that, under the current development of law, juvenile defendants have a right to effectively participate in the criminal (juvenile justice) procedures in which they are involved. The possibility of effective participation is an important cornerstone in building the complex constructs of the child’s procedural rights and child-friendly justice. Regarding this, Liefaard identifies three elements that ought to be taken into account in making access to justice child-friendly or “child-sensitive”: (1) child-friendly information; (2) child participation in proceedings; and (3) child-friendly remedies [10] (p. 216). We consider that quality legal aid might help to respond to all those needs; however, the most important role it plays is in strengthening effective participation. The instrument of individual assessment facilitates selecting child-friendly remedies but also, if implemented and used correctly, it allows the professionals to understand the child better and help him to participate in the proceedings more effectively.

States have developed and are continuing to improve various tools to address the situation of children in conflict with the law. Part of those tools are designed to assist the child and are seen as guarantees of his/her procedural rights, such as the child’s right to consult a lawyer, receive legal aid, or have an individual assessment carried out. The other part is modelled with an intention to change the child’s future behavior, strengthen his/her social and practical skills, reintegrate him/her into society, and enable the child to continue his/her life as a law-respecting citizen. While such tools are often seen as two separate building blocks of the criminal justice system, we consider that such an approach is misleading and there is a very strong inter-linkage between them. In particular, we believe that these procedural guarantees correspond to the right to legal aid and individual assessment (if implemented in interdisciplinary teams focusing on the quality of their services), enable the child’s effective participation in the proceedings, and create the foundation for the success of later reintegration programs.

The findings of the research projects presented in the paper show that, while professionals recognize the importance of effective children participation, many obstacles must be overcome to make participation more than just a formal aspiration. As for legal aid, although legal aid providers strive to develop trusting relationships with their young clients, communicate with them in a child-friendly manner, and thus become the child’s spokesman and guarantor of his rights, it is clear that there is a need to strengthen competencies of legal aid professionals and to improve existing practice and working conditions in order to do so. Similarly, professionals see advantages of individual assessment of the child; however, there are many challenges in implementing this tool in practice. They range from a lack of experience with this relatively new tool to the need for further instruments which can specify the scope of such assessment. For instance, professionals implementing individual assessments believe that specific unified instruments, toolkits, or guidelines on how to involve children effectively considering their views and opinions could solve their major problems. However, it is doubtful whether additional instruments are the top priority; we believe that strengthening capacities of professionals working with children, changing their approach toward children protection, and improving working conditions are more important. Policymakers, legal and non-legal professionals, and other stakeholders should be made aware of the importance of hearing children’s voices, considering their opinion, and making their participation meaningful.

## Data Availability

The data supporting reported results can be found: https://teise.org/en/lti-veikla/projektines-veiklos/teisine-pagalba-vaikams-baudziamajame-procese-gerosios-praktikos-formavimas-ir-sklaida-la-child/ (accessed on 1 November 2021) and https://teise.org/en/lti-veikla/projektines-veiklos/ia-child/ (accessed on 1 November 2021).
